# Clinical and epidemiological profiles of burns from a regional burn center in Egypt

**DOI:** 10.1038/s41598-026-48318-4

**Published:** 2026-05-04

**Authors:** Mohamed H. Elshahidi

**Affiliations:** Resident, Burns and Plastic Surgery Center, Mansoura University Hospitals, Mansoura, Dakahlia, Egypt

**Keywords:** Burn epidemiology, Mortality, Egypt, Pediatric burns, ICU admission, Diseases, Health care, Medical research, Risk factors

## Abstract

Burns impose a disproportionate burden on low- and middle-income countries, but epidemiological data are limited. Between 1 January and 31 December 2025, a prospective study was undertaken at a regional burn center in Mansoura, Dakahlia, Egypt, including all emergency admissions. The study cohort comprised 125 patients; 68.8% were male and 52.8% were pediatric (< 18 years). Burns showed bimodal seasonal peaks in winter and summer. Burns involving 10–19% of total body surface area (TBSA) were most common, accounting for 56% of cases. Mean (± SD) TBSA was 11% ± 7% in pediatric patients and 20% ± 21% in adults. Male gender, accidental etiology, and domestic occurrence predominated. Scalds were the most frequent mechanism of injury, followed by flame and electrical burns. Delayed admission beyond six hours post-injury occurred in 32% of cases. Adults had a longer mean hospital stay (25 ± 20 days) compared with pediatric patients (20 ± 16 days), whereas pediatric patients constituted the majority of those requiring skin grafting. Overall mortality was 11.2%, primarily among adults. In multivariate logistic regression, an ABSI score ≥ 7 was independently associated with mortality (AOR = 20.926 (95% CI: 3.355–130.52), *p* = 0.001). For ICU admission, an ABSI score ≥ 7 and TBSA > 25% were a statistically significant predictor. The pediatric cohort in this study demonstrated a heightened vulnerability to burn injuries, emphasizing the pivotal role of caregiver supervision and targeted preventive education. Conversely, mortality rates were disproportionately higher within the adult cohort.

**Supplementary Information:**

The online version contains supplementary material available at 10.1038/s41598-026-48318-4.

## Introduction

A burn injury is a multifaceted traumatic event to the external protective barrier of the human body, triggering cascading pathophysiological responses. Freezing, flames, scalds, electricity, radiation, or hot objects may cause varying degrees of coagulative tissue necrosis depending on the rate of energy transfer. In 2021, the global incidence of burns affecting more than 20% of the total body surface area (TBSA) was estimated to be 12.99 million cases, with an anticipated increase of 233.4% by the year 2050^1^. In the United States, burns are the fourth leading cause of death from trauma, with 40,000 hospitalizations and 3275 estimated deaths annually^2^. In 2023, the mean daily cost of burn care in the United States was estimated at US$8,844, underscoring the substantial financial burden imposed on healthcare systems and highlighting the need for effective resource planning and allocation strategies^3^.

Although burn injuries can affect anyone, at any time and in any place, 95% of the burdens occur in low- and middle-income countries (LMICs)^4^. While a declining trend in burn incidence has been observed in high-income countries, the magnitude of the burden in LMICs remains insufficiently recognized and is disproportionately underrepresented in the published scientific literature^5^. This situation leads to a consequential gap in our collective knowledge towards burn injury prevention and management^6^. LMICs lack standardized surveillance and data collection systems, which impede accurate characterization of the spatiotemporal patterns and trends of burn injuries^7^. Though burns constitute a significant public health concern when they occur, the majority are preventable through targeted interventions emphasizing public awareness, education, and the implementation of safety measures^8^. In 2024, a systematic review of burn injuries in the Middle East and North Africa (MENA) region reported that burns predominantly affected males, were most commonly accidental in nature, and were associated with a mean total body surface area (TBSA) involvement of 17.2% and an average hospital length of stay of 11.8 days^9^.

In Egypt, the available literature on burn epidemiology is limited, with only a few studies published between 1997 and 2025 addressing the epidemiological and clinical profile of burn injuries. In a 2024 study from a regional university burn center, scald injuries predominated, total body surface area (TBSA) involvement was 10–20%, and the majority of patients were pediatric, with a mean age of 2.3 years among children and 30 years among adults^10^. In African studies, flame burns, age less than 5 years, and TBSA > 20% have been identified as significant factors associated with burn-related mortality. The mortality rate has been reported to reach 17%^11,12^.

Egypt, with an estimated population of 116.5 million, represents the most populous state in the MENA region and the Arab world, and ranks third in population among African nations^13^. Located in the Nile Delta, Dakahlia ranks as Egypt’s fourth largest governorate, with a population of approximately 7 million. Situated in the capital city of the governorate, the Mansoura Burn Center is affiliated with the governorate’s primary university. The available literature on burn epidemiology in Egypt is largely based on retrospective studies, most of which have been conducted in major metropolitan areas such as Cairo and Alexandria. Furthermore, some investigations have concentrated on specific subpopulations, particularly pediatric patients. Despite these contributions, epidemiological data from Egypt remain relatively scarce, with only a limited number of published reports, including a study describing cases from the Mansoura Burn Center^14^.

This study aims to identify the demographics, clinical characteristics and outcomes of patients with burns admitted to the Mansoura Burn Center.

## Methods

### Study design

This study was conducted as a prospective, observational cohort investigation from January 2025 to December 2025, encompassing patients admitted to the Mansoura Burn Center via the Mansoura Emergency Hospital, Dakahlia, Egypt. The study was reported in accordance with the STROBE (Strengthening the Reporting of Observational Studies in Epidemiology) guidelines for observational research^15^.

### Study participants

Inclusion criteria for this study were established based on the Emergency Management of Severe Burns (EMSB) referral criteria^16^. Exclusion criteria included Stevens–Johnson syndrome, toxic epidermal necrolysis syndrome, prior prolonged management at another center, outpatient admissions, and transfers for planned advanced surgical interventions.

### Variables and outcomes

Upon admission to Mansoura Emergency Hospital, patients underwent initial assessment by plastic surgery residents. Those meeting the inclusion criteria were subsequently transferred to the burn center for definitive management. Data collection encompassed demographic characteristics, injury-related variables, total body surface area burned (TBSA), associated comorbidities, anatomical distribution of injuries, the need for escharotomy or amputation, requirement for skin grafting, and mortality risk. Burn severity was evaluated using two validated prognostic scoring systems: the Abbreviated Burn Severity Index (ABSI) score and the Revised Baux score^17^.

### Statistical analysis

The study population was stratified according to age into adult and pediatric groups (age < 18 years), and according to outcome into survivors and non-survivors. Categorical variables were presented as absolute frequencies and corresponding percentages, whereas continuous variables were summarized using the mean and standard deviation (SD), as well as the median and interquartile range (IQR). Data tabulation and analysis were performed using IBM SPSS Statistics for Windows, Version 28 (IBM Corp., Armonk, N.Y., USA, 2021). Associations between categorical variables and the study groups were evaluated using the chi-square (χ²) test. The categorical variables analyzed included gender, occupation, residence, manner of injury, place of injury, season and month of injury, mortality, intensive care unit (ICU) admission, escharotomy, amputations, need for skin grafting, and need for central venous catheter (CVC) insertion. When the assumptions of the chi-square test were not met, specifically when the expected cell count was less than 5 in any contingency table cell, Fisher’s exact test was applied as an alternative. The Mann–Whitney U test was applied to compare continuous variables between two independent groups (age, total body surface area [TBSA], revised Baux score and length of hospital stay). For comparisons involving three or more independent groups (age categories, TBSA strata, degree of burn, type of burn, ABSI and delay in presentation), the Kruskal–Wallis test was used.

The Least Absolute Shrinkage and Selection Operator (LASSO) technique, implemented via L1-penalized regression, was first applied to select variables for inclusion in the multivariable (adjusted) analysis, thereby reducing the risk of over-fitting. This technique was performed using the *glmnet* package, *R* (version 4.5.3)^18^.

Setting alpha = 1 with 5-fold cross-validation, the *cv.glmnet* function was then executed to identify the optimal regularization parameter, lambda (λ). The *lambda.min* value corresponds to the λ that minimizes the cross-validated error. Clinically relevant variables associated with mortality and ICU admission were considered in the LASSO model. Variables with non-zero coefficients were retained and included in the adjusted multivariable logistic regression analysis. Variables selected for LASSO model and their results are presented in the supplementary files. Receiver operating characteristic (ROC) curves were plotted, and the area under the curve (AUC) with corresponding 95% confidence intervals was calculated. Details of variable selection and the assessment of multicollinearity and ROC and AUC data are provided in the supplementary files. Subsequently, a multivariable logistic regression model was fitted to estimate adjusted odds ratios (AORs) with 95% confidence intervals (CIs), in order to identify independent factors significantly associated with mortality and ICU admission^19^. Following LASSO variable selection, multicollinearity among the retained variables was assessed using variance inflation factors (VIF). Variables with VIF values < 5 indicate no significant multicollinearity.

Statistical significance was defined as a two-sided *P-value* of < 0.05.

### Ethical considerations

The study adhered to relevant guidelines and regulations in accordance with the Declaration of Helsinki. Participants and/or their legal guardians were fully informed of the study objectives, methodology, and anticipated benefits. Ethical approval was obtained from the Institutional Review Board of Mansoura Faculty of Medicine (Ethics Code: R.25.01.3011). Oral informed consent was secured from all patients and/or their legal guardians prior to study enrollment and data collection.

## Results

**Fig. 1:**
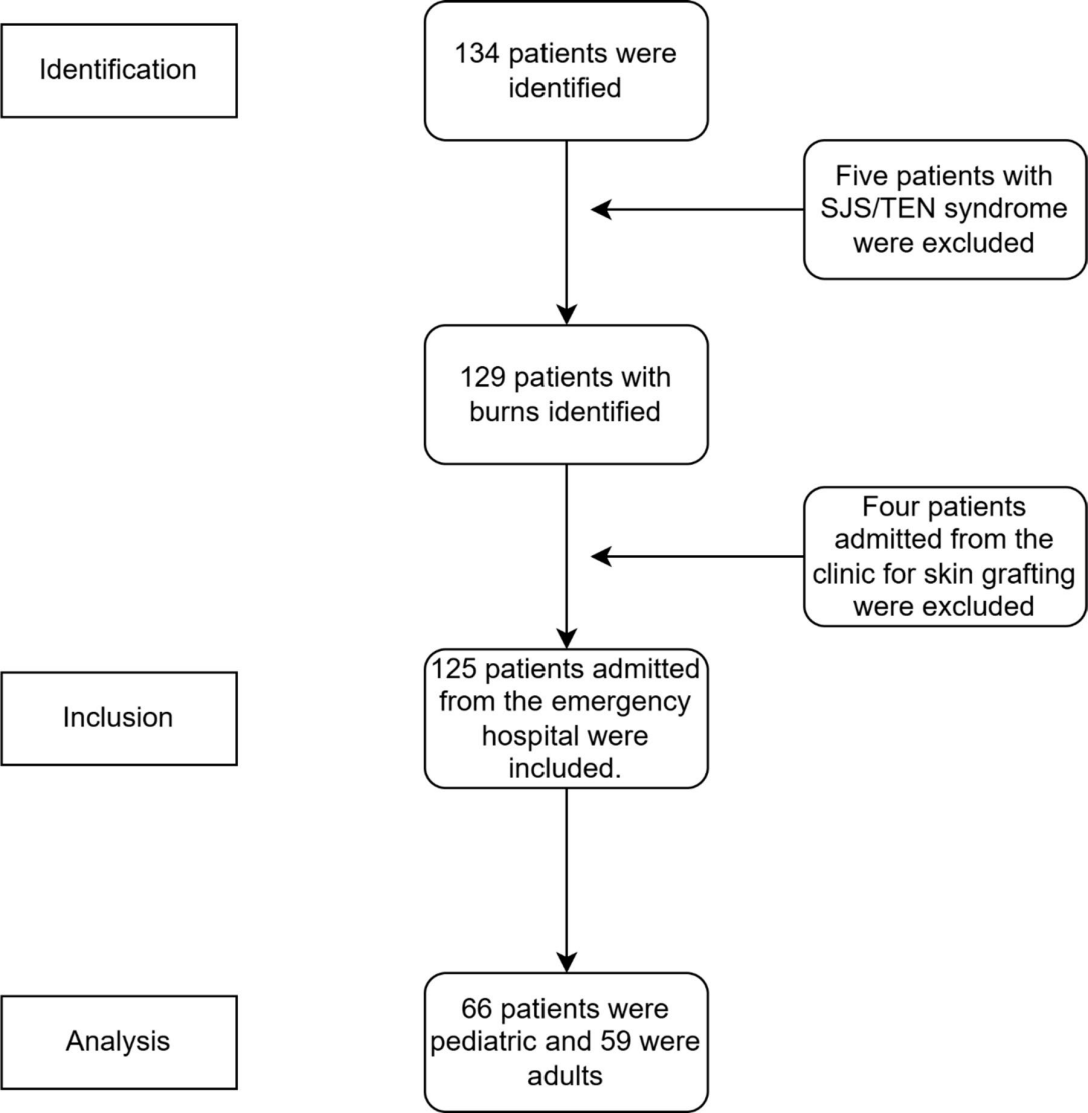
Flow-chart showing the number of participants assessed for eligibility, excluded, enrolled, and analyzed for the primary outcome.

A total of 134 patients were admitted to the Mansoura Burn Center, of whom five were diagnosed with Stevens–Johnson syndrome/toxic epidermal necrolysis (SJS/TEN) syndrome or severe drug reactions, and four were admitted for routine management or elective skin grafting from the outpatient clinic [Figure 1].

The study cohort comprised 125 patients, including 66 pediatric cases (52.8%; mean age 5.8 ± 4.9 years) and 59 adult cases (47.2%; mean age 41.9 ± 16.7 years) [Table 1].

**Table 1:** Summary characteristics of the study cohort.

No.	Total	Pediatric	Adult	*P*-value
	125 (100%)	66 (52.8%)	59 (47.2%)	
Age	Mean (± SD)	5.79 (4.92)	41.92 (16.69)	< 0.001
	Median (IQR)	3.25 (8.5)	40 (24)	
Age Group	0–9	49 (74.2%)	0	< 0.001
	10–19	17 (25.8%)	3 (5.1%)	
	20–29	0	14 (23.7%)	
	30–39	0	11 (18.6%)	
	40–49	0	11 (18.6%)	
	50–59	0	11 (18.6%)	
	60–69	0	3 (5.1%)	
	≥ 70	0	6 (10.2%)	
TBSA Group	0–9%	19 (28.8%)	10 (16.9%)	0.008
	10–19%	40 (60.6%)	30 (50.8%)	
	20–29%	3 (4.5%)	7 (11.9%)	
	30–39%	4 (6.1%)	3 (5.1%)	
	40–49%	0	3 (5.1%)	
	≥ 50%	0	6 (10.2%)	
Gender	Female	16 (24.2%)	23 (39%)	0.085
	Male	50 (75.8%)	36 (61%)	
Occupation	Infant	9 (13.6%)	0	< 0.001
	Child	30 (45.5%)	0	
	Student	26 (39.4%)	3 (5.1%)	
	House wife	0	17 (28.8%)	
	Engineer	0	3 (5.1%)	
	Military personnel	0	1 (1.7%)	
	Teacher	0	4 (6.8%)	
	Worker	0	26 (44.1%)	
	Senior	0	5 (8.5%)	
Residence	Rural	30 (45.5%)	24 (40.7%)	0.253
	Urban	32 (48.5%)	35 (59.3%)	
	Bedouin	3 (4.5%)	0	
	Refugee	1 (1.5%)	0	
TBSA	Mean (SD)	11.02% (± 7.23)	20.36% (± 20.56)	0.006
	Median (IQR)	10% (10)	10% (15)	
	Scald	46 (69.7%)	13 (22%)	< 0.001
	Flame	16 (24.2%)	37 (62.7%)	
	Electrical	2 (3%)	6 (10.2%)	
	Chemical	2 (3%)	1 (1.7%)	
	Contact	0	2 (3.4%)	
Degree	Superficial Second	35 (53%)	20 (33.9%)	0.006
	Deep Second	29 (43.9%)	28 (47.5%)	
	Third	2 (3%)	11 (18.6%)	
Manner	Accidental	65 (98.5%)	56 (94.9%)	0.469
	Homicidal	1 (1.5%)	1 (1.7%)	
	Suicidal	0	2 (3.4%)	
Site	Home	52 (78.8%)	34 (57.6%)	0.026
	School	1 (1.5%)	0	
	Street	8 (12.1%)	14 (23.7%)	
	Work	5 (7.6%)	11 (18.6%)	
Time to presentation	Mean (SD)	13.76 (23.46)	19.07 (64.16)	0.98
	Median (IQR)	3 (22)	3 (8)	
	0–6 h	43 (65.2%)	42 (71.2%)	0.482
	6–12 h	5 (7.6%)	5 (8.5%)	
	12–24 h	10 (15.2%)	5 (8.5%)	
	> 24 h	8 (12.1%)	7 (11.9%)	
Season	Winter	20 (30.3%)	22 (37.3%)	0.799
	Spring	14 (21.2%)	12 (20.3%)	
	Summer	18 (27.3%)	16 (27.1%)	
	Autumn	14 (21.2%)	9 (15.3%)	
Month	January	5 (7.6%)	2 (3.4%)	0.23
	February	5 (7.5%)	4 (6.8%)	
	March	11 (16.7%)	18 (30.5%)	
	April	3 (4.5%)	2 (3.4%)	
	May	6 (9.1%)	4 (6.8%)	
	June	5 (7.6%)	4 (6.8%)	
	July	11 (16.7%)	4 (6.8%)	
	August	2 (3%)	7 (11.9%)	
	September	7 (10.6%)	6 (10.2%)	
	October	3 (4.5%)	5 (8.5%)	
	November	4 (6.1%)	0	
	December	4 (6.1%)	3 (5.1%)	
Length of stay	Mean (± SD)	20.41 (± 16.4)	24.8 (± 20.35)	0.434
	Median (IQR)	14 (17)	17 (30)	
Mortality		2 (3%)	12 (20.3%)	0.002
Inhalational Injury		5 (7.6%)	9 (15.3%)	0.256
ICU admission		16 (24.2%)	19 (32.2%)	0.425
Escharotomy		1 (1.5%)	7 (11.9%)	0.026
Amputation		0	4 (6.8%)	0.047
Skin grafting		20 (30.3%)	12 (20.3%)	0.224
Need for CVC		8 (12.1%)	15 (25.4%)	0.056
ABSI score	Mean (± SD)	2.74 (± 0.86)	5.5 (± 2.37)	< 0.001
	Median (IQR)	3 (1)	5 (3)	
Revised Baux score	Mean (± SD)	18.09 (± 9.43)	64.86 (± 26.57)	< 0.001
	Median (IQR)	15.95 (11.75)	58 (30)	

### Age

**Fig. 2:**
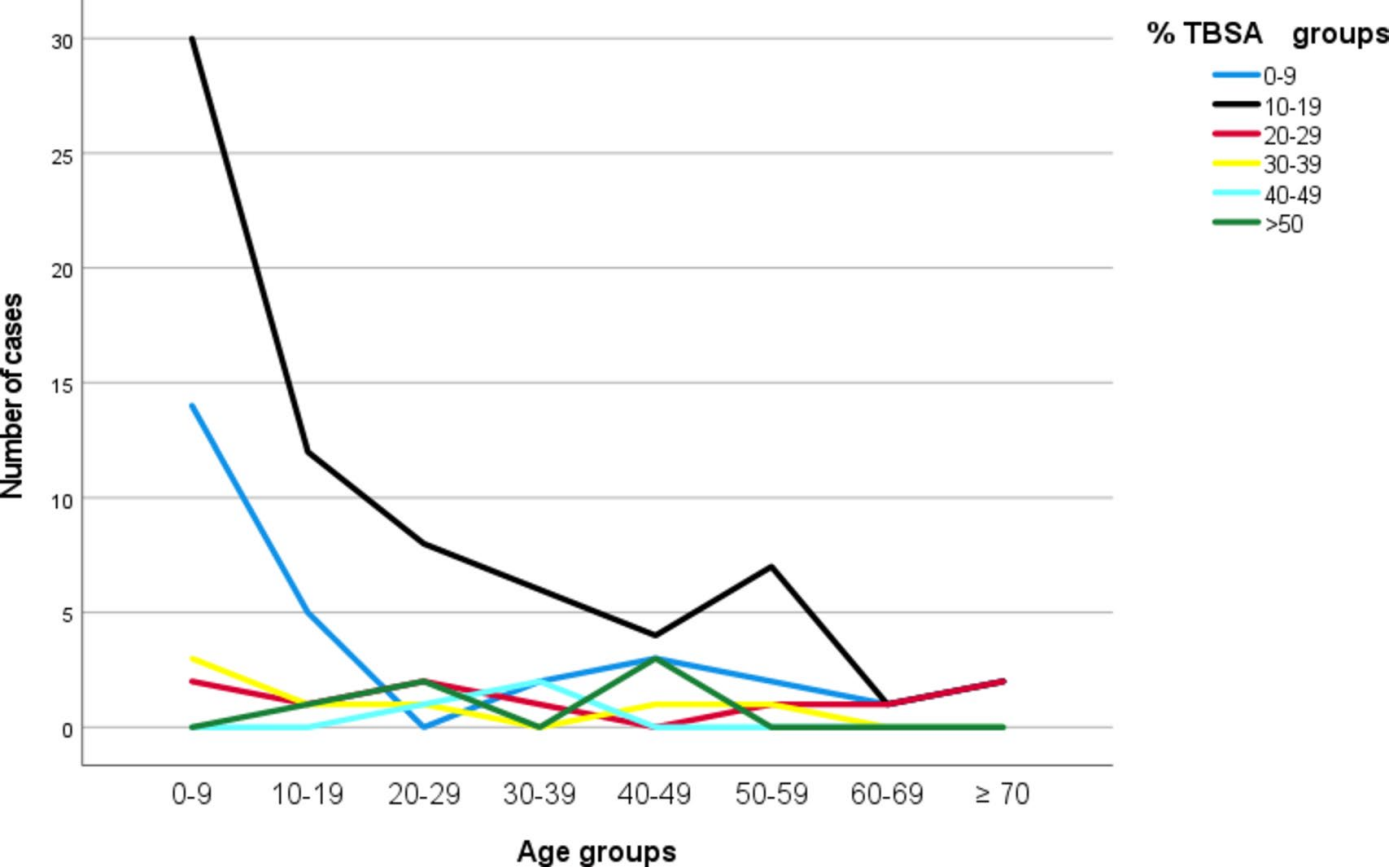
Distribution of study cohort in relation to age and TBSA.

Within the pediatric group, 49 patients (74.2%) were aged 0–9 years, whereas 42 patients (71.2%) in the adult group were aged 30 years or older. Survivors were significantly younger than non-survivors, with median ages of 12 (IQR: 32) and 39.5 years (IQR: 37.25), respectively (*p* = 0.021) [Tables 1 and 2]. Among individuals aged < 20 years, burns involving < 25% TBSA were the most common [Figure 2].

**Table 2:** Summary of study variables by clinical outcome: survivors and non-survivors.

No.	Total	Survivors	Non-survivors	*P*-value
	125 (100%)	111 (88.8%)	14 (11.2%)	
Age	Median (IQR)	12 (32)	39.5 (37.25)	0.021
	Mean (± SD)	20.94 (**±** 20.71)	37.83 (**±** 24.25)	
Age Group	0–9	47 (42.3%)	2 (14.3%)	0.009
	10–19	19 (17.1%)	1 (7.1%)	
	20–29	11 (9.9%)	3 (21.4%)	
	30–39	10 (9%)	1 (7.1%)	
	40–49	8 (7.2%)	3 (21.4%)	
	50–59	10 (9%)	1 (7.1%)	
	60–69	2 (1.8%)	1 (7.1%)	
	≥ 70	4 (3.6%)	2 (14.3)	
TBSA Group	0–9%	29 (26.1%)	0	< 0.001
	10–19%	68 (61.3%)	2 (14.3%)	
	20–29%	7 (6.3%)	3 (21.4%)	
	30–39%	6 (5.4%)	1 (7.1%)	
	40–49%	1 (0.9%)	2 (14.3%)	
	≥ 50%	0	6 (42.9%)	
Gender	Female	36 (32.4%)	3 (21.4%)	0.546
	Male	75 (67.6%)	11 (78.6%)	
Occupation	Infant	7 (6.3%)	2 (14.3%)	0.012
	Child	30 (27%)	0	
	Student	28 (25.2%)	1 (7.1%)	
	House wife	15 (13.5%)	2 (14.3%)	
	Engineer	2 (1.8%)	1 (7.1%)	
	Military personnel	1 (0.9%)	0	
	Teacher	3 (2.7%)	1 (7.1%)	
	Worker	22 (19.8%)	5 (35.7%)	
	Senior	3 (2.7%)	2 (14.3%)	
Residence	Rural	50 (45%)	4 (28.6%)	0.542
	Urban	57 (51.4%)	10 (71.4%)	
	Bedouin	3 (2.7%)	0	
	Refugee	1 (0.9%)	0	
TBSA	Median (IQR)	10% (10)	45% (40)	< 0.001
	Mean (± SD)	11.47% (± 7.4%)	46.79% (± 26.5%)	
	Scald	56 (50.5%)	3 (21.4%)	0.09
	Flame	42 (37.8%)	11 (78.6%)	
	Electrical	8 (7.2%)	0	
	Chemical	3 (2.7%)	0	
	Contact	2 (1.8%)	0	
Degree	Superficial Second	53 (47.7%)	2 (14.3%)	< 0.001
	Deep Second	51 (45.9%)	6 (42.9%)	
	Third	7 (6.3%)	6 (42.9%)	
Manner	Accidental	109 (98.2%)	12 (85.7%)	0.023
	Homicidal	2 (1.8%)	0	
	Suicidal	0	2 (14.3%)	
Site	Home	77 (69.4%)	9 (64.3%)	0.653
	School	1 (0.9%)	0	
	Street	20 (18%)	2 (14.3%)	
	Work	13 (11.7%)	3 (21.4%)	
Time to presentation	Mean (± SD)	13.05 (± 22.66)	41.79 (± 126.70)	0.52
	Median (IQR)	3 (11)	3.5 (10)	
	0–6 h	77 (69.4%)	8 (57.1%)	0.569
	6–12 h	6 (5.4%)	4 (28.6%)	
	12–24 h	15 (13.5%)	0	
	> 24 h	13 (11.7%)	2 (14.3%)	
Season	Winter	36 (32.4%)	6 (42.9%)	0.888
	Spring	24 (21.6%)	2 (14.3%)	
	Summer	30 (27%)	4 (28.6%)	
	Autumn	21 (18.9%)	2 (14.3%)	
Month	January	5 (4.5%)	2 (14.3%)	0.687
	February	8 (7.2%)	1 (7.1%)	
	March	26 (23.4%)	3 (21.4%)	
	April	4 (3.6%)	1 (7.1%)	
	May	9 (8.1%)	1 (7.1%)	
	June	9 (8.1%)	0	
	July	14 (12.6%)	1 (7.1%)	
	August	7 (6.3%)	2 (14.3)	
	September	12 (10.8%)	1 (7.1%)	
	October	6 (5.4%)	2 (14.3%)	
	November	4 (3.6%)	0	
	December	7 (6.3%)	0	
Length of stay	Mean (SD)	23.11 (**±** 17.47)	17.50 (**±** 25.02)	0.008
	Median (IQR)	17 (21)	7.5 (18)	
ICU admission		21 (18.9%)	14 (100%)	< 0.001
Inhalational Injury		8 (7.2%)	6 (42.9%)	0.001
Escharotomy		3 (2.7%)	5 (35.7%)	< 0.001
Amputation		3 (2.7%)	1 (7.1%)	0.382
Skin grafting		30 (27%)	2 (14.3%)	0.249
Need for CVC		12 (10.8%)	11 (78.6%)	< 0.001
ABSI score	Mean (± SD)	3.53 (± 1.4)	8.42 (± 2.92)	< 0.001
	Median (IQR)	3 (2)	8 (4.5)	
	2–3	62 (55.9%)	1 (7.1%)	
	4–5	38 (34.2%)	1 (7.1%)	
	6–7	11 (9.9%)	2 (14.3%)	
	8–9	0	6 (42.9%)	
	10–11	0	1 (7.1%)	
	≥ 12	0	3 (21.4%)	
Revised Baux score	Mean (SD)	33.64 (22.44)	91.9 (36.45)	< 0.001
	Median (IQR)	26 (33)	100 (57.75)	

### Gender

Overall, males accounted for 86 patients (68.8%), with a male-to-female ratio of 3.21:1 in the pediatric subgroup and 1.56:1 in the adult subgroup. Among deceased patients, males predominated with a male-to-female ratio of 3.66:1, although the difference was not statistically significant (*p* = 0.546).

### Manner of injury

The predominant mechanism of injury was accidental, accounting for 121 cases (96.8%), whereas suicidal and homicidal injuries were each observed in 2 cases (1.6%).

### Aetiology of burns

**Fig. 3:**
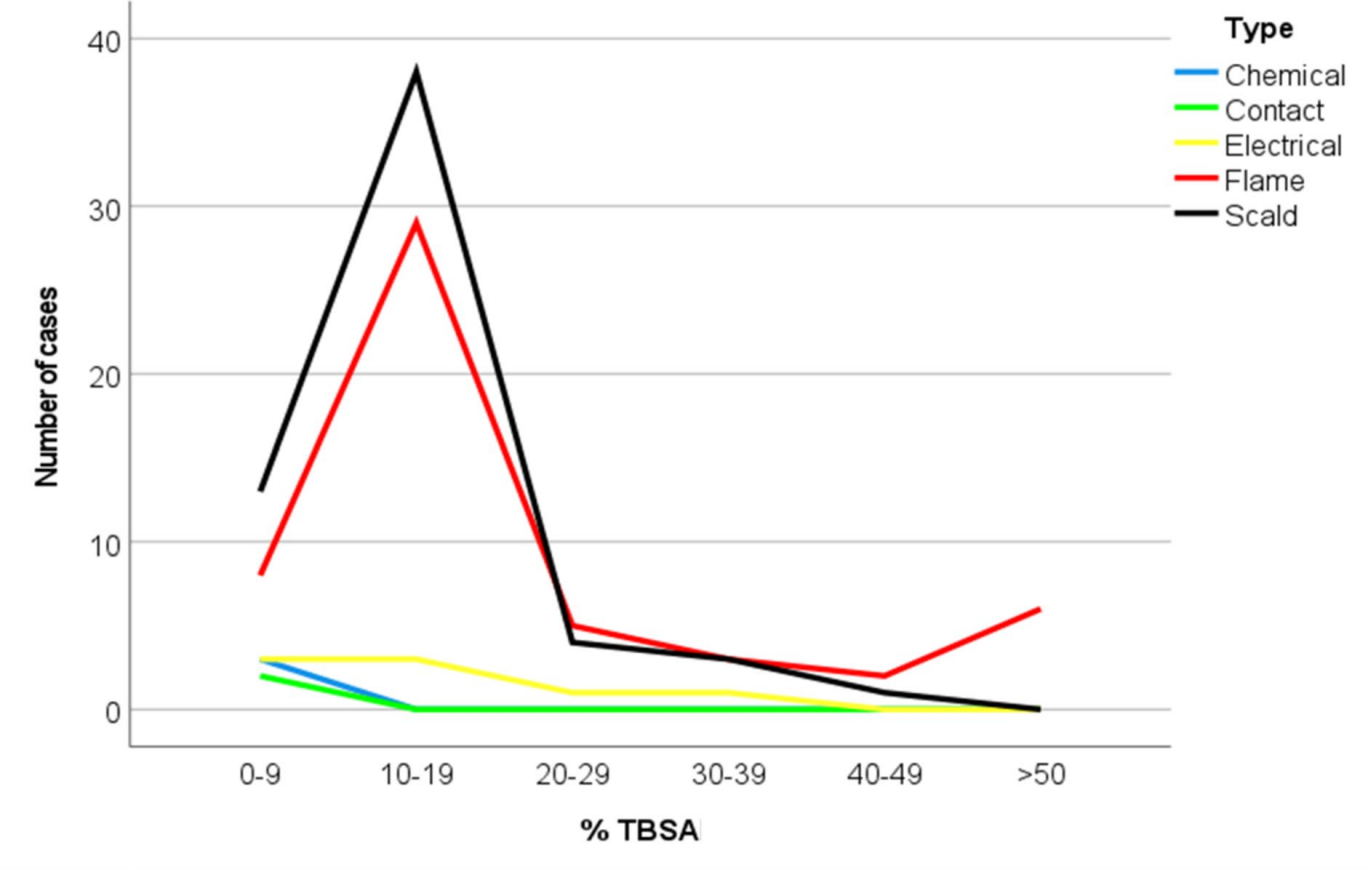
TBSA and aetiology of burn injury.

The most common mode of burn injury was scalding, observed in 59 cases (47.2%) primarily within the pediatric cohort, while flame burns affected 53 cases (42.4%), mainly in the adult cohort. Electrical burn injuries were observed in 8 cases (6.4%), predominantly in adults, whereas chemical burns occurred in 3 cases (2.4%), mainly among pediatric patients. In burns involving TBSA < 20%, scald and flame injuries represented the principal etiologies; conversely, major burns were characterized by a relatively uniform distribution across different causative mechanisms [Figure 3].

### Prehospital delay

Pediatric patients presented to the emergency department after a mean interval of 14 (± 23) hours, compared with 19 (± 64) hours among adults; overall, 32% of patients presented later than 6 h post-injury.

### Scene of injury

Most injuries occurred in the domestic setting (86, 68.8%), while occupational injuries accounted for 16 cases (12.8%).

### Temporal trend

**Fig. 4:**
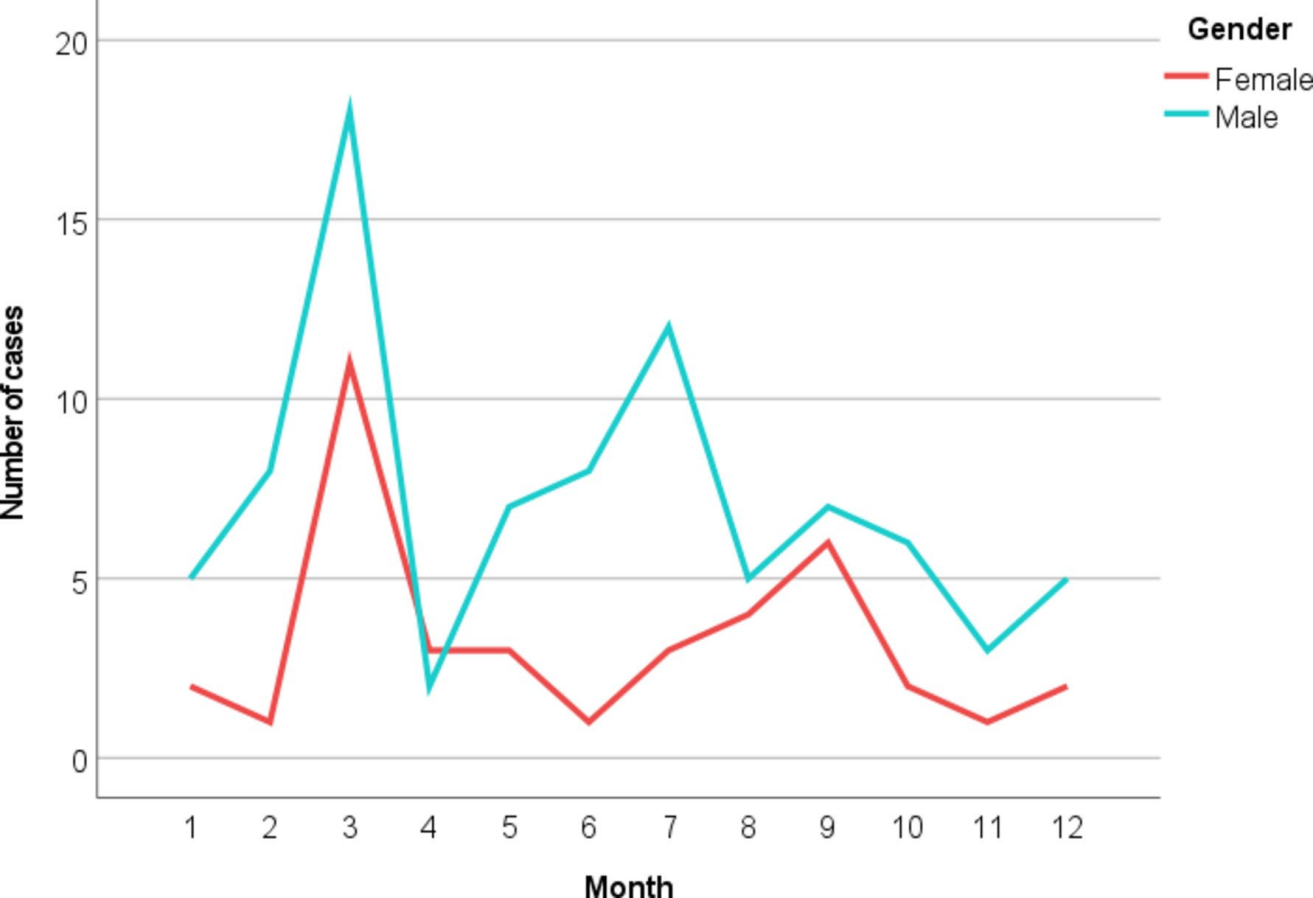
Monthly distribution of cases stratified by gender.

Burn injuries exhibited a bimodal seasonal pattern, with peaks in winter (42 cases, 33.6%) and summer (34 cases, 27.2%). Winter predominance was slightly greater among adults, whereas summer predominance was marginally higher among pediatric patients [Figure 4].

### Topographical distributions of injuries

**Fig. 5:**
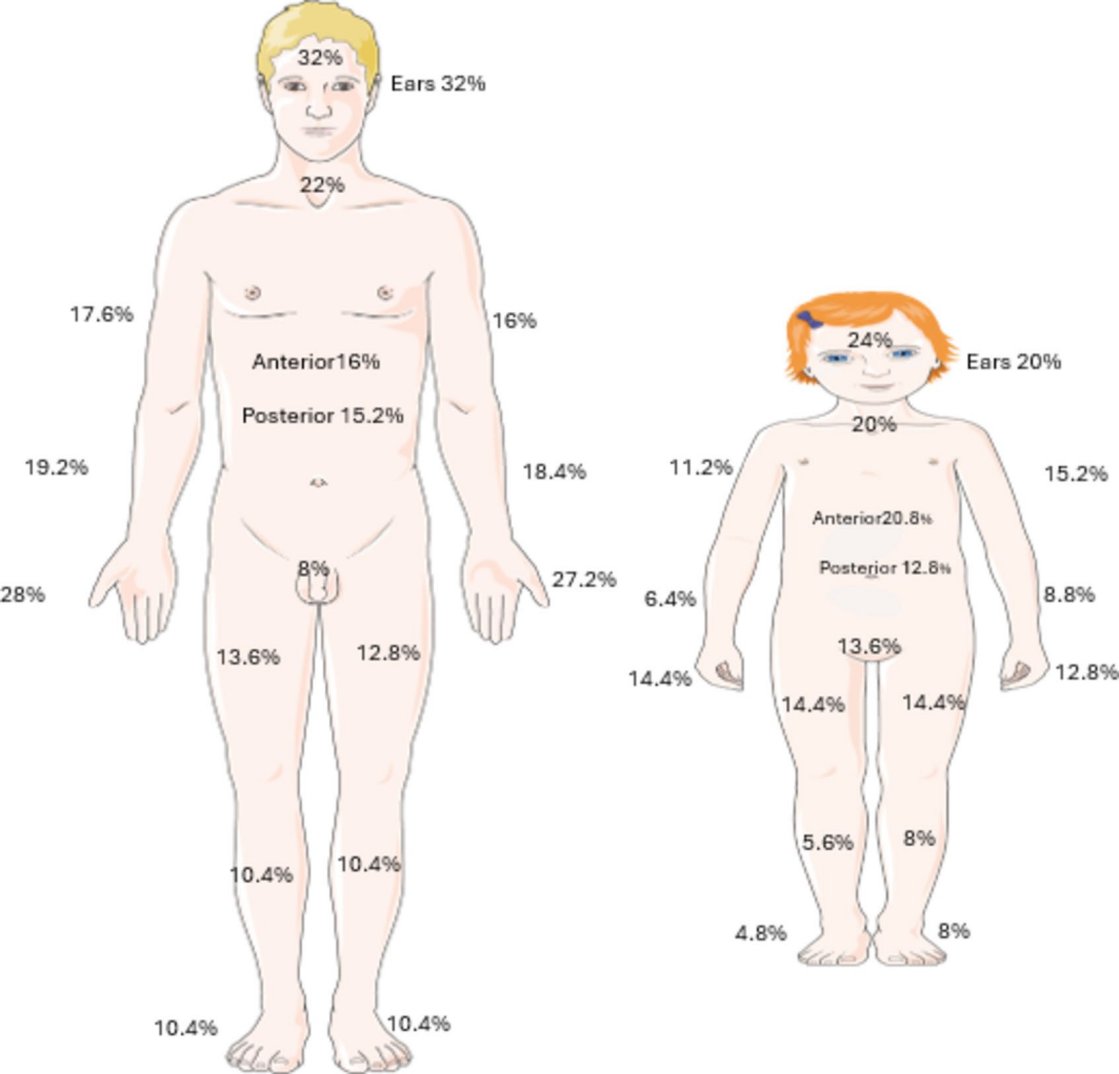
Proportion of anatomical regions affected in adult versus pediatric populations. Image(s) provided by Servier Medical Art (https://smart.servier.com), licensed under CC BY 4.0 (https://creativecommons.org/licenses/by/4.0/).

Burn involvement of the head, arms, forearms, and hands was more frequent among adults than pediatric patients, whereas perineal involvement was more commonly observed in the pediatric cohort [Figure 5].

### Total body surface area (TBSA) burned

**Fig. 6:**
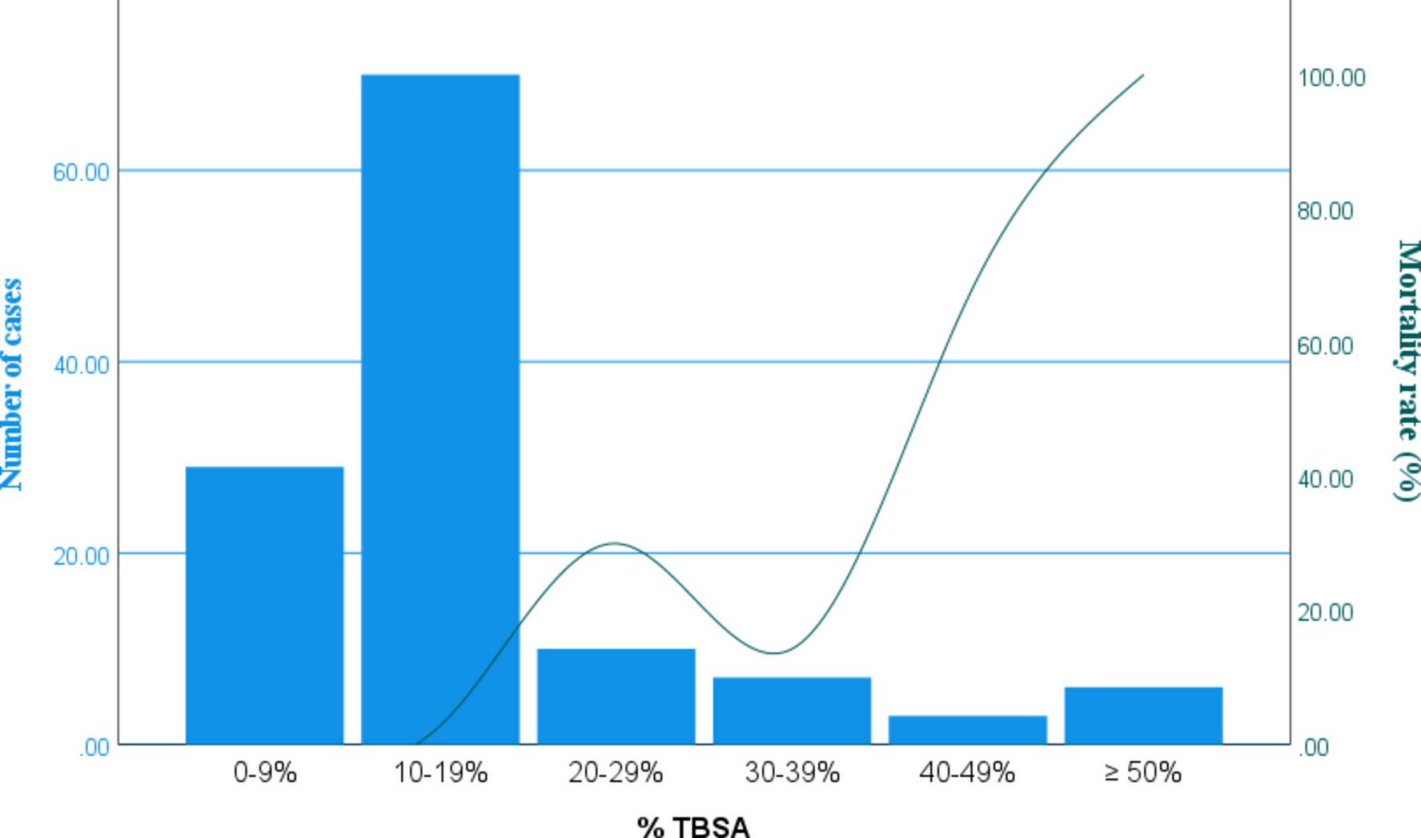
Mortality rate stratified by TBSA.

Pediatric patients had a mean TBSA of 11% ± 7%, compared with 20% ± 21% in adults. Overall, nonfatal burns involving < 20% TBSA constituted the majority of cases (99, 79.2%) [Figure 6].

### Burn depth

Within the cohort, second-degree burns constituted the majority of cases (112, 89.6%), with a disproportionately higher occurrence among pediatric patients (64, 51.2%) than adults (48, 38.4%). Upon admission, full-thickness burns were more frequently encountered in the adult cohort (11 cases, 8.8%) than in pediatric patients (2 cases, 1.6%).

### Length of hospital stay (LOS)

**Fig. 7:**
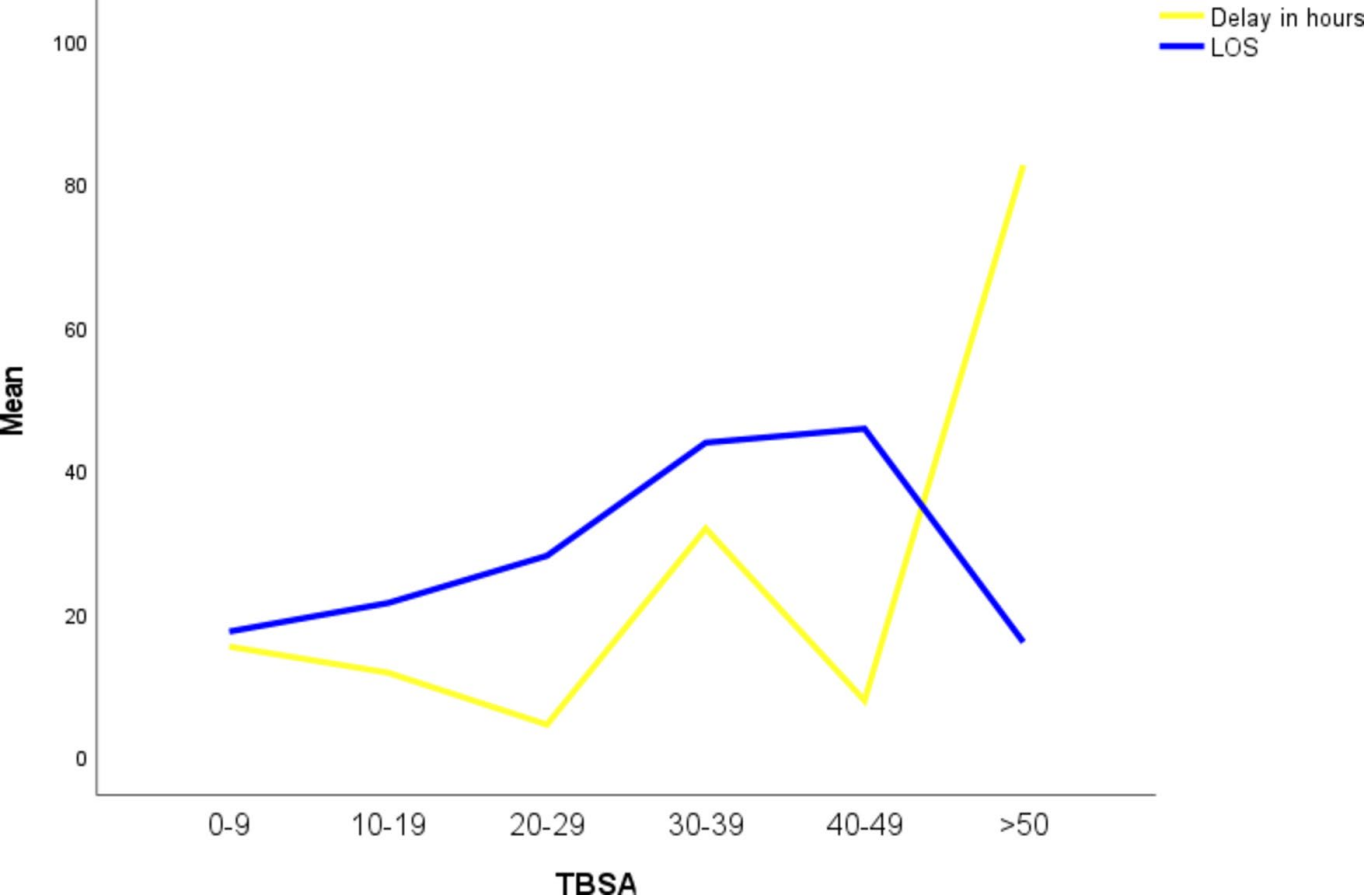
Delay in presentation ( in hours) and length of hospital stay (in days) stratified by TBSA.

Adults experienced a marginally longer mean LOS (25 ± 20 days) than pediatric patients (20 ± 16 days). Length of stay increased proportionally with escalating TBSA up to 40–49%; however, beyond this threshold, the highest mortality rates were observed, likely influencing hospitalization duration [Figure 7].

### Mortality

**Fig. 8:**
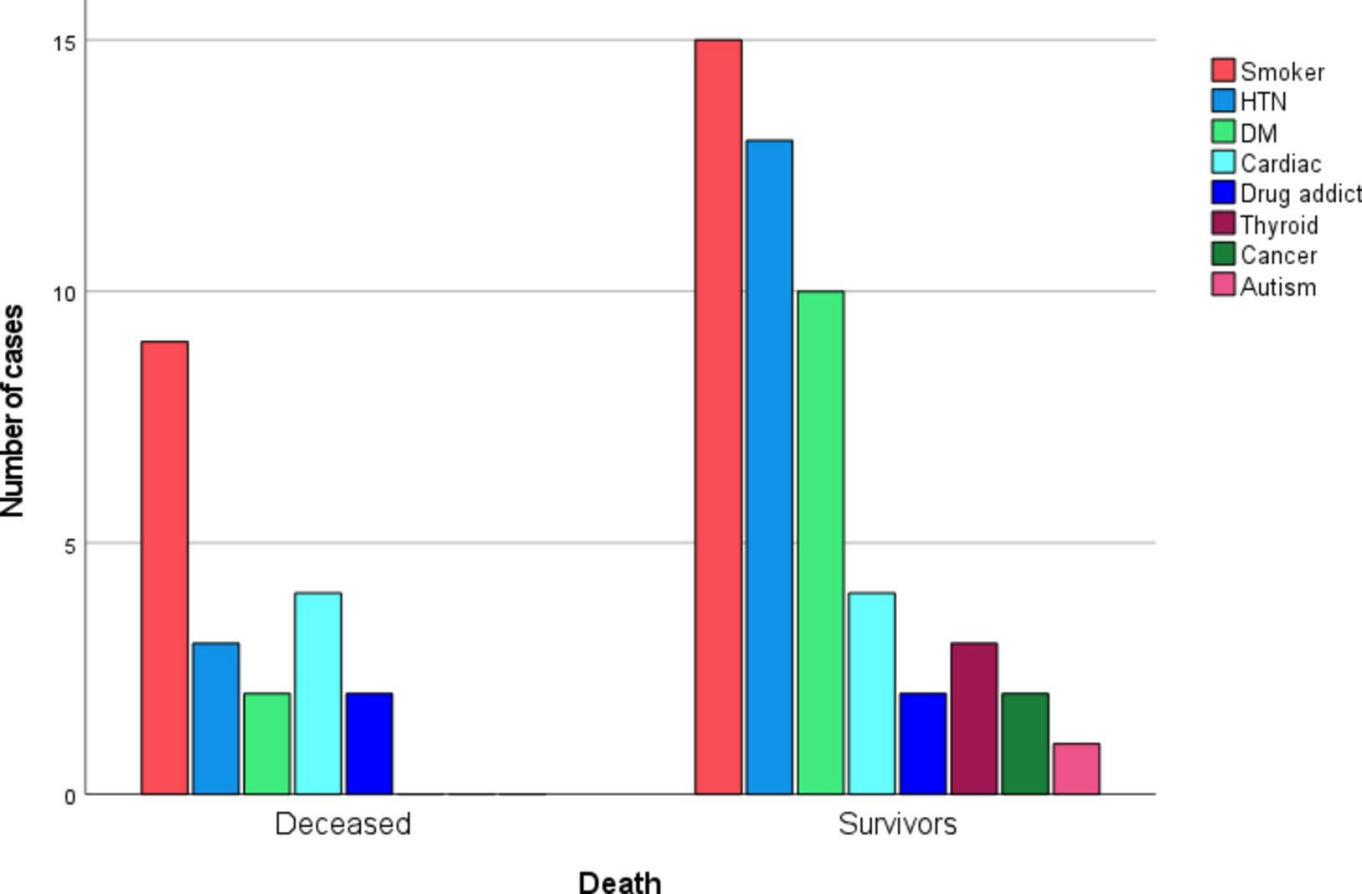
Prevalence of co-morbid conditions among survivors and non-survivors groups.

Overall mortality in the study cohort was 14 cases (11.2%). Mortality was higher among adults, with 12 deaths among 59 adult patients (20.3%), compared with 2 deaths among 66 pediatric patients (3.0%). In addition, mortality demonstrated a steep escalation once TBSA exceeded 40% [Figure 6]. A greater prevalence of co-morbid conditions—smoking, hypertension, diabetes mellitus, and substance abuse—was observed among patients who did not survive [Figure 8]. Multivariate logistic regression analysis was performed to identify factors associated with the primary outcome (mortality) (Table 3). An ABSI score ≥ 7 demonstrated the strongest association with mortality, followed by central venous catheter (CVC) use, TBSA > 25%, smoking status, third-degree burn depth, and inhalational injury. In contrast, hypertension (HTN), diabetes mellitus (DM), gender, and delay in presentation were not significantly associated with mortality. In the adjusted multivariate logistic regression analysis, only ABSI score ≥ 7 demonstrated a statistically significant association with mortality [Table 4].

**Table 3:** Logistic regression analysis (unadjusted) of predictors of mortality.

Variable	Unadjusted odds ratio (OR)	95% CI	*p*-value
ABSI ≥ 7	64.167	14.052-293.012	< 0.001
CVC usage	30.25	7.384-123.026	< 0.001
TBSA > 25%	26.743	7.041–101.57	< 0.001
Smoking	11.52	3.397–39.066	< 0.001
Third degree	11.143	3.018–41.14	< 0.001
Inhalational injury	9.656	2.685–34.723	0.001
Adult	8.17	1.745–38.247	0.008
Flame	6.024	1.588–22.844	0.008
HTN	2.056	0.506–8.350	0.314
Male gender	1.760	0.462–6.702	0.407
Delay > 6 h	1.699	0.547–5.274	0.359
DM	1.683	0.329–8.608	0.532
Revised Baux score	1.069	1.038–1.101	< 0.001
Age	1.032	1.008–1.057	0.009
LOS	0.98	0.944–1.017	0.288
Skin grafting	0.45	0.095–2.13	0.314
TBSA < 25%	0.037	0.10-0.142	< 0.001

**Table 4:** Logistic regression analysis (adjusted) of predictors of mortality. Nagelkerke R-square = 0.57.

Variable	Adjusted odds ratio (OR)	95% CI	*p*-value
ABSI ≥ 7	20.926	3.355–130.520	0.001
TBSA > 25%	4.741	0.751–29.931	0.098
Third degree burns	3.249	0.462–22.866	0.237

To assess the applicability of the predictive model, receiver operating characteristic (ROC) curve analysis was conducted. The area under the curve (AUC) values were 0.866 (95% confidence interval [CI]: 0.736–0.995) for ABSI ≥ 7, 0.790 (95% CI: 0.635–0.945) for TBSA > 25%, and 0.683 (95% CI: 0.510–0.856) for third-degree burns. These differences were statistically significant.

### Clinical outcomes

Admission to the intensive care unit (ICU) was required in 35 cases (28%), of which pediatric patients accounted for 16 cases (45.7%). A total of 32 patients (25.6%) underwent skin grafting, with pediatric patients accounting for 20 cases (62.5%) of these procedures. Escharotomy was indicated in 8 cases (6.4%), predominantly in adults, who accounted for 7 cases (87.5%). In the multivariate logistic regression model evaluating predictors of ICU admission, only an ABSI score ≥ 7 and TBSA > 25% demonstrated a statistically significant association [Table 5]. Within the ICU admission predictive model, the area under the curve (AUC) values were 0.663 (95% CI: 0.547–0.780) for ABSI ≥ 7, 0.649 (95% CI: 0.532–0.766) for TBSA > 25%, and 0.550 (95% CI: 0.434–0.666) for hypertension. ABSI ≥ 7 and TBSA > 25% were statistically significant predictors of ICU admission, whereas hypertension did not reach statistical significance.

**Table 5:** Multivariate logistic regression analysis of ICU admission. Nagelkerke R-square = 0.268.

Variable	Adjusted odds ratio (AOR)	95% CI	*p*-value
TBSA > 25%	5.479	1.23–24.4	0.026
ABSI ≥ 7	4.606	1.056–20.09	0.042
Hypertension	2.128	0.61–7.418	0.236

## Discussion

In this study, the epidemiological characteristics of burn injuries and their associated morbidity and mortality risks among patients presenting to a large regional referral center were evaluated. In recognition of the anatomical and physiological differences between children and adults, the study cohort was divided into pediatric (< 18 years) and adult (≥ 18 years) groups. Compared with adults, children have thinner skin with fewer dermal appendages and a proportionally larger body surface area–to–mass ratio, factors that predispose them to more extensive burn injury and reduced healing capacity, often necessitating skin grafting^20^. In a 2021 analysis of the World Health Organization’s Global Burn Registry (GBR), pediatric patients accounted for 42% of burn cases, with a mean age of 5.3 years. In the present study, children comprised a higher proportion of cases (52.8%) with a mean age of 5.8 years, reflecting an increased pediatric representation relative to global data^21^. In addition, males predominated in this burn center cohort, consistent with global trends. However, gender disparities in burn injuries and outcomes have been reported in the literature, with females sustaining more severe burns and exhibiting higher in-hospital mortality. These disparities are particularly pronounced in low- and middle-income countries^22^.

Also, accidental burns and those occurring within the home constitute the majority of cases, underscoring the critical need for injury prevention strategies and the implementation of effective in-house safety measures. The etiology of burn injuries exhibits an age-dependent pattern, with scalds predominating in the pediatric cohort and flame burns in adults. In this cohort, delayed hospital admission (> 24 h) was observed in nearly 12% of cases. The literature suggests that factors such as substance use and poor social support networks increase the risk of delayed presentation, which is further associated with a greater need for intensive care^23^. The bimodal seasonal peak observed in winter and summer in this cohort has been similarly reported in diverse geographic regions worldwide^24^. Furthermore, the increased incidence of pediatric burns during the summer months may be attributed to reduced supervision during outdoor activities and the use of lighter, less protective clothing. This study cohort also demonstrated a predominance of burns involving < 20% TBSA, consistent with well-established epidemiological patterns reported across diverse geographic regions worldwide^25^.

In addition, reported mortality in this study cohort corresponds closely to rates documented in Western populations as well as in MENA countries^9,26^. Although smoking is widely acknowledged to compromise wound healing, the literature demonstrates variable associations between smoking status and clinical outcomes in burn patients^27,28^. In addition, growing evidence indicates that substance use exacerbates burn severity, leading to prolonged hospitalization, increased ICU requirements, and elevated mortality risk^29^. Furthermore, the study cohort demonstrated that although pediatric patients predominantly present with second-degree burns, the majority of cases requiring skin grafting occur in this age group. The Abbreviated Burn Severity Index (ABSI), which includes gender, age, total body surface area (TBSA), inhalation injury, and the presence of full-thickness burns, showed a statistically significant association with mortality in the adjusted multivariate logistic regression analysis. An ABSI score ≥ 7 emerged as a practical and reliable cut-off threshold of mortality prediction. Furthermore, the AUC values for ABSI, TBSA, and burn severity were statistically significant, supporting their robustness as predictors of mortality risk among patients admitted to our center. This finding is consistent with reports from different regions of the world, including China and Switzerland^30,31^.

In addition, an ABSI score ≥ 7 and increased total body surface area (TBSA) > 25% were significantly associated with ICU admission in the adjusted multivariate regression analysis. These results are consistent with findings reported from countries with varying socio-economic backgrounds, with the exception of the association with age^32^. Furthermore, although the length of hospital stay observed in this cohort aligns with previous reports from Egypt, it was longer than that reported in studies from upper-middle-income countries^33,34^. The prolonged hospital stay observed in this cohort may, in part, be attributable to inhalation injury, increased burn depth, the requirement for multiple surgical procedures, and unstable clinical conditions necessitating ICU care. Although previous studies have demonstrated that burn wound infection significantly predicts total LOS, this factor was not evaluated in the current study and therefore its impact cannot be ascertained^35^.

Despite these findings, the results should be interpreted cautiously due to several limitations. The study included only patients presenting to the emergency department, excluding those managed in outpatient settings. As a tertiary referral center, the Mansoura Burn Center receives transferred cases from other hospitals, potentially introducing a referral bias toward more severe burns. Additionally, the study was conducted at a single center over a 12-month period, limiting temporal and geographic representativeness. Furthermore, the small cohort size and limited number of mortality events may compromise the statistical power of the logistic regression analysis. Finally, data on burn wound infections and antimicrobial resistance were not collected. Collectively, these factors may influence the reported burn severity and limit the generalizability of the findings to the wider burn population in Egypt.

## Conclusion

Epidemiological studies of burn injuries are essential for quantifying the magnitude of this public health problem. They enable the identification of risk factors and the characterization of high-risk populations. Such investigations also provide critical insights into patterns of morbidity and mortality. Importantly, they generate the evidence necessary to guide public awareness initiatives and inform the allocation of healthcare resources aimed at mitigating the impact of this devastating form of trauma. Given the paucity of data regarding burn injuries in Egypt, this study, conducted at a large regional center, provides valuable insights into the temporal trends of such injuries within the region. However, the relatively short study period and the limited sample size necessitate caution in generalizing these findings to the broader population.

## Electronic Supplementary Material

Below is the link to the electronic supplementary material.

Supplementary Material 1

Supplementary Material 2

Supplementary Material 3

## Data Availability

All data generated or analyzed during this study are included. The datasets used and/or analyzed during the current study are available from the corresponding author upon reasonable request.

## References

[1] Lee, N., Bae, Y., Jang, S., Lee, D. W. & Lee, S. W. Global, Regional, and National Burden of Burn Injury by Total Body Surface Area (TBSA) Involvement from 1990 to 2021, with Projections of Prevalence to 2050. *Healthcare* 13, (2025). 10.3390/healthcare13162077 PMC12385682 40868692

[2] Burns https://www.sciencedirect.com/science/chapter/monograph/abs/pii/B978032354947900122X10.1016/B978-0-323-54947-9.00122-X

[3] Malek, K., Zhao, T., Lou, R., Ozhathil, D. & Khandelwal, A. 613 Analysis of Cost, Patient Charges and Reimbursement at a Regional Burn Center. *J. Burn Care Res.* **46**, S187 (2025). 10.1093/jbcr/irag140 42593749

[4] Peck, M. D. Epidemiology of burns throughout the world. Part I: Distribution and risk factors. *Burns J. Int. Soc. Burn Inj*. **37**, 1087–1100 (2011). 10.1016/j.burns.2011.06.005 21802856

[5] Khushalani, A. & Srivastava, S. The epidemiology of burns in a tertiary care center of Rajasthan. *Int. Surg. J.* **10**, 1484–1489 (2023).

[6] Kneib, C. J. et al. 602 Too Few Burn Papers and Images, Lowering Publication Barriers for Low- and Middle-Income Country Authors. 10.1093/jbcr/irae036.236

[7] Davé, D. R., Nagarjan, N., Canner, J. K., Kushner, A. L. & Stewart, B. T. Rethinking burns for low & middle-income countries: Differing patterns of burn epidemiology, care seeking behavior, and outcomes across four countries. *Burns* **44**, 1228–1234 (2018). 29475744 10.1016/j.burns.2018.01.015

[8] McCann, C., Watson, A. & Barnes, D. Major burns: Part 1. Epidemiology, pathophysiology and initial management. *BJA Educ.* **22**, 94–103 (2022). 35211326 10.1016/j.bjae.2021.10.001 PMC8847805

[9] Elshahidi, M. H. Clinico-demographic profile of burns in the Middle-East and North-Africa (MENA) region: a systematic review and meta-analysis. *Discov Public. Health*. **21**, 154 (2024).

[10] Sadaka, M. S. & Abdeldaim, D. E. Study of the Relationship Between Patient Demographics, Burn Etiology, and the Incidence of Burn Wound Infection in Tanta University Burn Unit. *Ann. Burns Fire Disasters*. **37**, 3–9 (2024). 38680837 PMC11041824

[11] Belayneh, A. G. et al. Treatment outcome and associated factors of burn injury in Ethiopian hospitals: A systematic review and meta-analysis. *Scars Burns Heal*. **11**, 20595131251321772 (2025). 10.1177/20595131251321772 PMC11909675 40092716

[12] Nthumba, P. M. Burns in sub-Saharan Africa: A review. *Burns J. Int. Soc. Burn Inj*. **42**, 258–266 (2016). 10.1016/j.burns.2015.04.006 25981292

[13] World Bank Open Data. *World Bank Open Data* https://data.worldbank.org

[14] El Hadidy, M. et al. A retrospective statistical analysis of burnt patients in the period between 2002–2006 in the Burn Unit of Mansoura University Hospitals–Egypt. *J. Plast. Reconstr. Surg.* **33**, 239–243 (2009).

[15] von Elm, E. et al. The Strengthening the Reporting of Observational Studies in Epidemiology (STROBE) statement: guidelines for reporting observational studies. *Lancet* **370**, 1453–1457 (2007). 18064739 10.1016/S0140-6736(07)61602-X

[16] Van Yperen, D. T. et al. Adherence to the emergency management of severe burns referral criteria in burn patients admitted to a hospital with or without a specialized burn center. *Burns* **47**, 1810–1817 (2021). 33707084 10.1016/j.burns.2021.02.023

[17] Usmani, A. et al. Prediction of Mortality in Acute Thermal Burn Patients Using the Abbreviated Burn Severity Index Score: A Single-Center Experience. *Cureus* **14**, e26161 (2022). 35891871 10.7759/cureus.26161 PMC9302604

[18] Friedman, J. H., Hastie, T. & Tibshirani, R. Regularization Paths for Generalized Linear Models via Coordinate Descent. *J. Stat. Softw.* **33**, 1–22 (2010). 20808728 PMC2929880

[19] Kwak, S. G. LASSO Regression Analysis: Applications in Dyslipidemia and Cardiovascular Disease Research. *J. Lipid Atheroscler*. **14**, 289–297 (2025). 41048602 10.12997/jla.2025.14.3.289 PMC12488800

[20] Barrios, E. L. et al. Variables Influencing the Differential Host Response to Burns in Pediatric and Adult Patients. *Shock Augusta Ga.* **59**, 145–154 (2023). 36730790 10.1097/SHK.0000000000002042 PMC9957807

[21] Jordan, K. C., Di Gennaro, J. L., von Saint André-von Arnim, A. & Stewart, B. T. Global trends in pediatric burn injuries and care capacity from the World Health Organization Global Burn Registry. *Front. Pediatr.* **10**, 954995 (2022). 35928690 10.3389/fped.2022.954995 PMC9343701

[22] Mehta, K. et al. Gender-based disparities in burn injuries, care and outcomes: A World Health Organization (WHO) Global Burn Registry cohort study. *Am. J. Surg.* **223**, 157–163 (2022). 34330521 10.1016/j.amjsurg.2021.07.041 PMC8688305

[23] Manasyan, A. et al. Factors associated with delayed admission to the burn unit: A major burn center’s experience. *Burns* **50**, 107288 (2024). 39447286 10.1016/j.burns.2024.107288

[24] Shmelev, A., Robinson, E., Quiroga, L. H., Hultman, C. S. & Asif, M. 559 Seasonal Variation in Hospitalizations for Burn Injuries. *J. Burn Care Res.* **41**, S120 (2020).

[25] Chen, L. et al. Development of a framework for managing severe burns through a 17-year retrospective analysis of burn epidemiology and outcomes. *Sci. Rep.* **11**, 9374 (2021). 33931691 10.1038/s41598-021-88507-x PMC8087787

[26] Foppiani, J. A. et al. A Meta-Analysis of the Mortality and the Prevalence of Burn Complications in Western Populations. *J. Burn Care Res.* **45**, 932–944 (2024). 38619135 10.1093/jbcr/irae064

[27] Klifto, K. M. et al. Impact of nicotine/smoking, alcohol, and illicit substance use on outcomes and complications of burn patients requiring hospital admission: systematic review and meta-analysis. *Burns J. Int. Soc. Burn Inj*. **46**, 1498–1524 (2020). 10.1016/j.burns.2019.08.003 31818513

[28] Knowlin, L., Stanford, L., Cairns, B. & Charles, A. The effect of smoking status on burn inhalation injury mortality. *Burns J. Int. Soc. Burn Inj*. **43**, 495–501 (2017). 10.1016/j.burns.2016.09.003 PMC5376368 27707642

[29] Rahbar Taramsari, M. et al. The Effect of Drug Abuse on Clinical Outcomes of Adult Burn Patients Admitted to a Burn Center in the North of Iran. *Bull. Emerg. Trauma.* **11**, 90–95 (2023). 37193010 10.30476/beat.2023.98282.1424 PMC10182718

[30] Christ, A. et al. Revalidating the prognostic relevance of the Abbreviated Burn Severity Index (ABSI): A twenty-year experience examining the performance of the ABSI score in consideration of progression and advantages of burn treatments from a single center in Vienna. *J. Plast. Reconstr. Aesthet. Surg.* **94**, 160–168 (2024). 38805847 10.1016/j.bjps.2024.04.041

[31] Li, H. et al. Epidemiology and outcome analysis of 6325 burn patients: a five-year retrospective study in a major burn center in Southwest China. *Sci. Rep.* **7**, 46066 (2017). 28383066 10.1038/srep46066 PMC5382583

[32] Esen, O. et al. The factors affecting mortality in intensive care unit of burn center. *J. Pak Med. Assoc.* **73**, 763–766 (2023). 37051979 10.47391/JPMA.1045

[33] Wardhana, A. & Winarno, G. A. Epidemiology And Mortality Of Burn Injury In Ciptomangunkusumo Hospital, Jakarta: A 5 Year Retrospective Study. *J. Plast. Rekonstr*. **6**, 234–242 (2019).

[34] AbdelWahab, M. E., Sadaka, M. S., Elbana, E. A. & Hendy, A. A. Evaluation of prognostic factors affecting lenght of stay in hospital and mortality rates in acute burn patients. *Ann. Burns Fire Disasters*. **31**, 83–88 (2018). 30374257 PMC6199018

[35] Matias, M., Nogueira, R., Vasconcelos, C. & Bexiga, J. Factors predicting burn unit length-of-stay. *Rev Bras. Cir. Plástica* **39**, 1–13 (1 AD)(2024).

