# Supplementary material for: Clinical and epidemiological profiles of burns from a regional burn center in Egypt
Source: Sci Rep. 2026 May 4;16:14164. doi: 10.1038/s41598-026-48318-4 (PMC13139414; doi:10.1038/s41598-026-48318-4)
Supplement: Supplementary file 1 — Supplementary Material 1 [file 41598_2026_48318_MOESM1_ESM.docx]

| Coefficients | | | |
| --- | --- | --- | --- |
| Model |  | Collinearity Statistics | |
|  |  | Tolerance | VIF |
|  | ABSI_eq_more_7 | .559 | 1.789 |
|  | TBSA_more_25 | .620 | 1.613 |
|  | Degree_third_others | .840 | 1.191 |
| a Dependent Variable: Survival_Death | | | |

| Model |  | Collinearity Statistics |  |
| --- | --- | --- | --- |
|  |  | Tolerance | VIF |
|  | HTN | .899 | 1.113 |
|  | ABSI_eq_more_7 | .560 | 1.785 |
|  | TBSA_more_25 | .580 | 1.725 |
| a Dependent Variable: ICU admission | | | |

Multicollinearity diagnostic test for predictors retained after LASSO variable selection in the multivariable logistic regression model. All VIF values < 5 indicate no significant multicollinearity.
