# Supplementary material for: Clinical and epidemiological profiles of burns from a regional burn center in Egypt
Source: Sci Rep. 2026 May 4;16:14164. doi: 10.1038/s41598-026-48318-4 (PMC13139414; doi:10.1038/s41598-026-48318-4)
Supplement: Supplementary file 2 — Supplementary Material 2 [file 41598_2026_48318_MOESM2_ESM.docx]

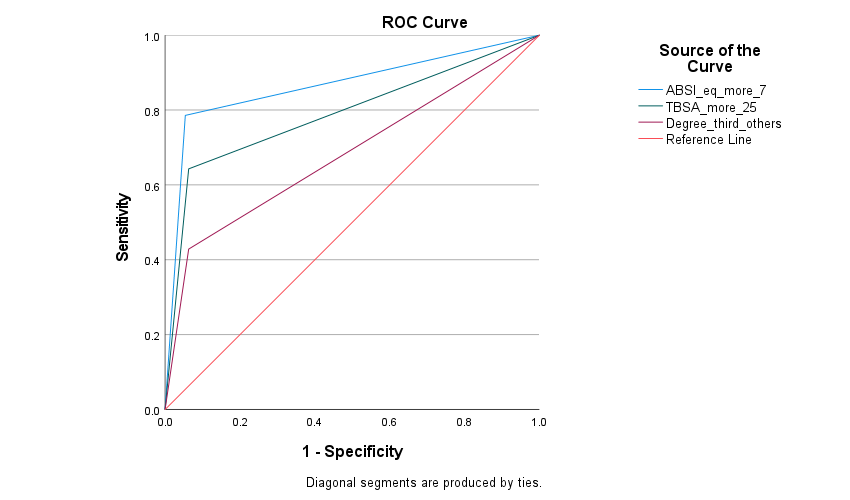


| **Area Under the Curve** | | | | | |
| --- | --- | --- | --- | --- | --- |
| Test Result Variable(s) | Area | Std. Error^a^ | Asymptotic Sig.^b^ | Asymptotic 95% Confidence Interval | |
|  |  |  |  | Lower Bound | Upper Bound |
| ABSI_eq_more_7 | .866 | .066 | .000 | .736 | .995 |
| TBSA_more_25 | .790 | .079 | .000 | .635 | .945 |
| Degree_third_others | .683 | .088 | .026 | .510 | .856 |
| The test result variable(s): ABSI_eq_more_7, TBSA_more_25, Degree_third_others has at least one tie between the positive actual state group and the negative actual state group. Statistics may be biased. | | | | | |
| a. Under the nonparametric assumption | | | | | |
| b. Null hypothesis: true area = 0.5 | | | | | |

Receiver operating characteristic curves (ROC) and analysis of the area under the curve (AUC) to predict mortality.


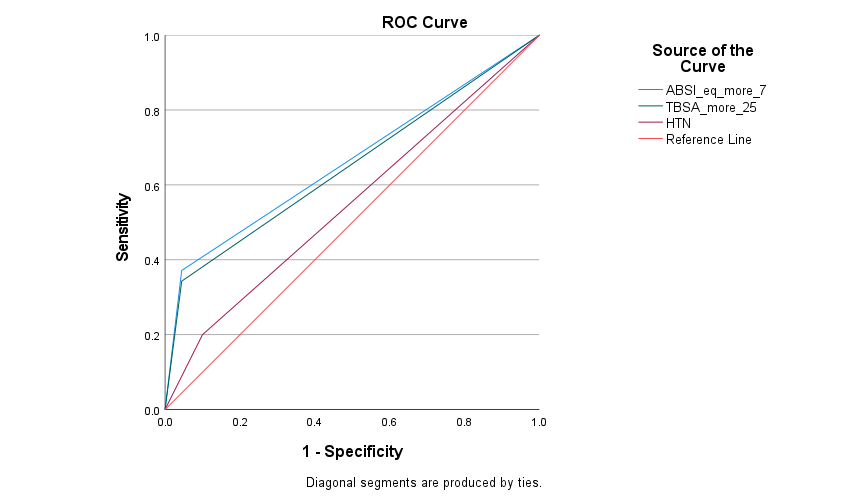


| **Area Under the Curve** | | | | | |
| --- | --- | --- | --- | --- | --- |
| Test Result Variable(s) | Area | Std. Error^a^ | Asymptotic Sig.^b^ | Asymptotic 95% Confidence Interval | |
|  |  |  |  | Lower Bound | Upper Bound |
| ABSI_eq_more_7 | .663 | .059 | .005 | .547 | .780 |
| TBSA_more_25 | .649 | .060 | .010 | .532 | .766 |
| HTN | .550 | .059 | .386 | .434 | .666 |
| The test result variable(s): ABSI_eq_more_7, TBSA_more_25, HTN has at least one tie between the positive actual state group and the negative actual state group. Statistics may be biased. | | | | | |
| a. Under the nonparametric assumption | | | | | |
| b. Null hypothesis: true area = 0.5 | | | | | |

Receiver operating characteristic curves (ROC) and analysis of the area under the curve (AUC) of predictive variables of ICU admission.
