# Supplementary material for: Clinical and epidemiological profiles of burns from a regional burn center in Egypt
Source: Sci Rep. 2026 May 4;16:14164. doi: 10.1038/s41598-026-48318-4 (PMC13139414; doi:10.1038/s41598-026-48318-4)
Supplement: Supplementary file 3 — Supplementary Material 3 [file 41598_2026_48318_MOESM3_ESM.docx]

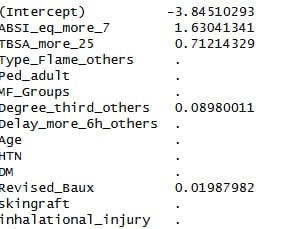


Variable considered for the LASSO analysis of mortality. Non-zero coefficients were selected for inclusion in the logistic regression analysis.


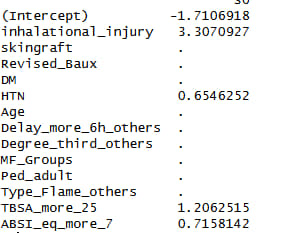


Variable considered for the LASSO analysis of ICU admission. Non-zero coefficients were selected for inclusion in the logistic regression analysis.
